# Suspected Transfusion Related Acute Lung Injury Improving following Administration of Tranexamic Acid: A Case Report

**DOI:** 10.1155/2014/710813

**Published:** 2014-06-04

**Authors:** Stan Ryniak, Piotr Harbut, Anders Östlund, Andrzej Mysiak, Jan G. Jakobsson

**Affiliations:** ^1^Department of Anaesthesia & Intensive Care, Karolinska Institutet, Danderyds University Hospital, Danderyd, 182 88 Stockholm, Sweden; ^2^Section for Trauma and Acute Anaesthesiology, Karolinska Institutet, Karolinska University Hospital, Danderyd, 182 88 Stockholm, Sweden; ^3^Department of Cardiology, Medical University, 50-367 Wroclaw, Poland

## Abstract

A 16-year-old woman with craniofacial injury developed severe acute respiratory failure under the primary reconstructive surgical procedure requiring several units of blood and plasma. A transfusion related acute lung injury (TRALI) was suspected and supportive treatment was initiated. Because of the severity of symptoms, acute extracorporeal membrane oxygenation (ECMO) was planned. During preparation for ECMO, a single intravenous dose, 1 g of tranexamic acid, was administered and a remarkable improvement was observed shortly thereafter. The patient was placed on ECMO for 16 hours. The further course was uncomplicated and the patient was discharged from ICU on the 6th day after admission fully and she recovered. A clinical improvement was observed in a timely fashion following the administration of tranexamic acid. The handling of a suspected TRALI and potential benefit from administration of tranexamic acid are discussed in this case report.

## 1. Introduction

Blood transfusion is considered safe when the infused blood is tested using state-of-the-art antigen-antibody and viral assays developed over the past several decades [1]. In recent years, transfusion reactions have however attracted attention as the list of potential nonhaemolytic complications from blood transfusion has grown [2].

We report a patient presenting a suspected severe transfusion related reaction causing profound respiratory compromise complicating the handling of multitrauma. The patient was successfully treated by the combination of supportive therapy, where the administration of protease inhibition, tranexamic acid 1 g iv., had a seemingly beneficial effect.

## 2. Case Report

**Figure 1:**
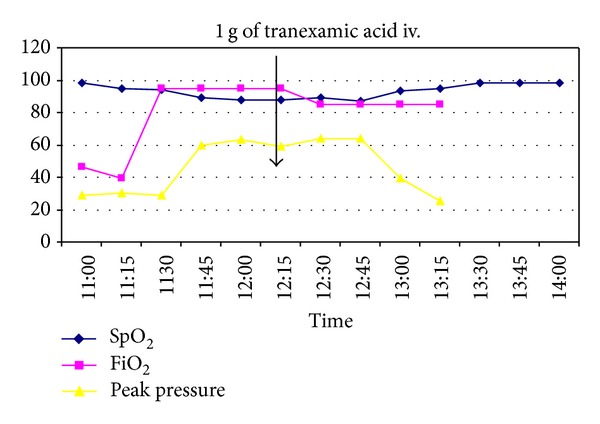
Clinical course: oxygen saturation, FiO_2_ and peak inspiratory pressure.

The patient has given a written informed consent around anonymous presentation of the clinical course, described as a case report. A 16-year-old woman was admitted to the Emergency Department of Karolinska University Hospital after a traffic accident, where she had been exposed to a craniofacial injury. According to the medical helicopter paramedics' report, she lost the consciousness for a short while; however, she had 15 in Glasgow Coma Scale (GCS) on admission and denied any previous medical history. Trauma CT did not show any intracranial pathology but possibly instable C5-arch fracture and dislocated right humerus fracture. The initial chest CT did not show any pathology, and the lungs were assessed as normal. Physical examination showed an extensive wound of right frontoparietal and temporal region causing severe bleeding with facial nerve injury. An acute surgical intervention was considered and the patient was anaesthetised by means of rapid sequence induction (RSI) and remained stable during and after the induction of general anaesthesia. No blood or other secretions were found in the airway during uncomplicated intubation. Removal of the facial compression dressings resulted in the increase of bleeding, which led to the transfusion of totally 8 units of packed red cells, 6 units of plasma, and 1 unit of platelets and the fact that haemoglobin (Hb) remained stable at around 10.0 g/dL level. One of the plasma units was from a female donor. About 45 minutes after the beginning of surgery sudden respiratory deterioration occurred, with an increase in oxygen demand up to FiO_2_ 0.95 as well as consecutive decrease in lung compliance with peak inspiratory pressure (PIP) reaching 60–65 cm H_2_O in order to maintain adequate ventilation (see Figure 1). Additionally, rush-like skin changes were observed. All the infusions were stopped and adrenaline/clemastine/cortisone was given based on the first hypothesis of an allergic reaction. Excessive plasma-like secretion from airway was observed some 5–10 minutes later, and repeated tracheobronchial suctioning was needed. Coexisting fast-progressive facial oedema was detected as well as Hb content rising up to 19.8 g/dL. The patient required circulatory support in form of continuous adrenaline infusion of 0.2 *μ*g/kg/min. A severe, life-threatening transfusion related acute lung injury (TRALI) was suspected as an alternative to an anaphylactic reaction. Because of the dramatic respiratory conditions, a decision was taken to start acute extracorporeal membrane oxygenation (ECMO) treatment. On awaiting the ECMO team, we administered a single intravenous dose 1 g of tranexamic acid for the purpose of possible protease inhibition in the pulmonary capillary circulation. Twenty to twenty-five minutes after tranexamic acid administration, during vascular cannulation for ECMO, a substantial improvement in respiratory mechanics as well as in oxygenation was observed. The patient presented an acceptable blood gas but still required FiO_2_ 0.85. ECMO treatment was started and continued in about 16 hours for lung protection reasons. The patient remained sedated and intubated for two days until the complementary facial nerve reconstruction was performed. She was subsequently weaned from ventilator within 48 hours and discharged from ICU on the sixth day after admission.

A number of diagnostic procedures were performed. Bedsides chest X-ray, thorax CT scans performed in conjunction with cannulation for ECMO showed changes typical for acute lung injury. Blood samples for tryptase and histamine were collected following initiation of ECMO and remained negative, which strongly refuted the cause for the severe reaction anaphylaxis. Granulocyte agglutination tests were performed in order to try to verify the diagnosis of TRALI but remained negative. Leukocyte antibodies were however found to be positive in one of the transfused plasma units, collected from a female donator, a finding that strengthens the diagnosis of TRALI.

## 3. Discussion

The improvements in blood product handling and testing for group compatibility have substantially reduced the occurrence of haemolytic reactions. Transfusion of tested blood products is today considered undramatic and reassuringly safe. Transfusion of blood products is however not without risk. The benefit versus risk must be assessed in each individual patient. There are rare but serious risks associated to blood component transfusion that must be kept in mind. Gilliss et al. published in 2011 an extensive review about the growing transfusions related concern and further efforts are indeed requested in order to reduce their occurrence and improve the handling of patients that show signs and symptoms in conjunction with administration of blood products [3]. Nonhaemolytic transfusion related reactions such as transfusion associated acute lung injury, circulatory overload, allergic reactions, febrile reactions, posttransfusion purpura, and graft-versus-host disease are uncommon types of transfusion reactions but must be acknowledged and recognized [4]. TRALI is a severe clinical syndrome with a wide variation in prevalence, ranging between 1 : 1323 and 1 : 1000000, depending on the diagnostic criteria and clinical manifestations. It is a condition where on the base of immunological reaction neutrophil sequestration and activation in pulmonary capillary circulation give rise to a massive protease release and subsequent sudden increase of pulmonary capillary permeability, leading to the pulmonary oedema with plasma leakage and acute hypoxic lung insufficiency. It is associated with an overall reported mortality ranging between 5% and 25%, usually lying in lower end, but it still remains the leading cause of transfusion-related deaths [2, 3]. Although transfusion related acute lung injury (TRALI) is in general considered a rare complication of transfusion medicine, the US Food and Drug Administration today acknowledges the syndrome as the leading cause of transfusion-related mortality [4]. The awareness of the problem within the medical society is still unsatisfactory which results in a high number of unrecognized cases or of inaccurate diagnoses, for example, cardiogenic pulmonary edema [5]. It affects receivers of all ages [6]. The mechanism that triggers TRALI is still a matter of discussion and there is no explicit recommendation for how to act in case of a suspected transfusion related acute lung reaction. Blood products' antibodies reaction with blood recipients' antigens leading to pulmonary neutrophil activation is the traditional explanation [7, 8]. However, there are studies that suggest the possibility of stepwise, two-event-“two-hit” mechanism. The first “trigger event” being associated with the underlying clinical condition causes a sequestration and activation of neutrophils in the pulmonary capillaries where the transfusion of antibody-reach blood products is the second trigger causing an extensive release of vasoactive mediators, for example, bradykinine.

Treatment of TRALI is today largely supportive and efforts are focusing on prevention [8, 9]. Since the pulmonary endothelium involvement and fenestration leading to an excessive plasma leakage resulting in hypoxia are the unquestionable endpoint of this syndrome, efforts aim at reducing and/or blocking the detrimental effects of protease activation. The administration of protease inhibitors could hypothetically influence the underlying mechanism and terminate the progress of lung injury [10, 11]. Tranexamic acid is one cascade blocker that is commonly available in the perioperative setting. We found in our patient an obvious time-related improvement after the tranexamic acid had been given. Tranexamic acid may be considered in severe bleed in conjunction with trauma as part of the general trauma care. Thus, one may argue that tranexamic acid should have been administered to our patients anyhow. Although debated the Cochrane review from 2012 suggests that tranexamic acid safely reduces mortality in bleeding trauma patients without increasing the risk of adverse events [12, 13]. There is of course a need for further data before any general recommendation for the use of tranexamic acid as part of the management of suspected TRALI.

There are without doubt other possible reasons for the severe respiratory insufficiency seen. We are not able to explicitly state a TRALI diagnosis. An allergic reaction was initially considered in the present case. Fluid overload and fat embolism are both possible alternative or additional explanations. From this single case, under dramatic clinical surgical care, it is not possible to draw any extensive conclusions. We still think it is of importance to share our experience first and most importantly to always have transfusion related acute lung injury in mind whenever massive blood and plasma transfusions are administered [14]. Recognition and awareness of the syndrome need to be highlighted among clinicians [15] and we secondly believe the use of tranexamic acid has a reasonable positive benefit risk profile in trauma patients showing signs of TRALI.
